# Case report: Spontaneous renal hemorrhage in anti-neutrophil cytoplasmic antibody-associated vasculitis

**DOI:** 10.3389/fimmu.2025.1544263

**Published:** 2025-01-29

**Authors:** Ruohan Yu, Lina Zhang, Ting Long, Hui Gao, Jing Xu, Tong Zhang, Shengguang Li

**Affiliations:** ^1^ Department of Rheumatology and Immunology, Peking University International Hospital, Beijing, China; ^2^ Department of Pathology, Peking University International Hospital, Beijing, China

**Keywords:** anti-neutrophil cytoplasmic antibody, ANCA-associated vasculitis, granulomatosis, polyangiitis, spontaneous renal hemorrhage, arterial aneurysm

## Abstract

Anti-neutrophil cytoplasmic antibody (ANCA)-associated vasculitis (AAV) is a systemic necrotizing vasculitis that predominantly affects small vessels. In this report, we present a typical case of granulomatosis with polyangiitis (GPA) complicated by spontaneous renal hemorrhage (SRH), a rare but potentially severe condition. The patient developed SRH during immunosuppressive therapy but recovered following conservative treatment. We then conducted a systematic literature review on SRH in the context of AAV, and analyzed clinical features, management strategies, and patient prognosis. A total of 15 patients were enrolled for statistical analysis, comprising the one case reported in the current study and 14 from the literature. Of these patients, nine presented with GPA and six showed microscopic polyangiitis (MPA), with a sex distribution of 3:2 males to females. The average patient age was 54.5 years, and ranged from 25 to 82 years. Acute flank pain was the most common clinical manifestation, and was occasionally accompanied by anemia and shock. Treatment varied for the different patients. Eight patients received glucocorticoid and immunosuppressive agents that included rituximab, cyclophosphamide, and azathioprine; five patients underwent transcatheter arterial embolization (TAE); and one patient underwent nephrectomy. Our findings indicate that SRH typically occurs early in the course of AAV and correlates with disease activity, with renal aneurysm rupture as the primary cause. More than half of the patients respond well to corticosteroids and immunosuppressants. Timely TAE is essential for patients showing persistent deterioration despite conservative management.

## Introduction

Anti-neutrophil cytoplasmic antibody (ANCA)-associated vasculitis (AAV) encompasses a group of systemic diseases that is characterized by necrotizing inflammation predominantly affecting small vessels, including granulomatosis with polyangiitis (GPA), microscopic polyangiitis (MPA), and eosinophilic granulomatosis with polyangiitis (EGPA) (1). According to the International Vasculitis Study Group, the global prevalence of AAV is estimated at approximately 100 cases per million individuals (2). Although primarily involving small vessels, the inflammatory process in AAV can extend to medium-sized vessels, which then leads to the formation and subsequent rupture of arterial aneurysms, and causes hemorrhage in affected organs (3).

Spontaneous renal hemorrhage (SRH) is a rare clinical condition that is characterized by acute, non-traumatic subcapsular and perirenal hematoma formation (4). Typical manifestations include acute flank pain, abdominal tenderness, and signs of internal bleeding (5). The most common etiologies are angiomyolipoma (42.2%), followed by vasculitis (15.7%) and malignancy (14.7%) (4). Among the etiology of vasculitis, polyarteritis nodosa (PAN) was the most commonly reported(75%) (4). Other vasculitides included Behcet’s disease and deficiency of adenosine deaminase 2 (DADA2) was also reported (6). Despite the numerous diseases that are associated with SRH, cases caused by AAV are only reported infrequently.

In this study, we not only presented a rare case of GPA complicated by SRH, but also conducted a systematic literature review of publications between 1 January 1990 and 1 April 2024 that concerned AAV associated with SRH. With this literature review we aimed to provide a comprehensive perspective on the epidemiology, clinical presentations, diagnostic challenges, and therapeutic strategies of SRH in AAV. We expect that our work will enhance clinicians’ awareness and management capabilities for this rare but severe complication.

## Case presentation

A 52-year-old male was admitted to our Rheumatology Department with a 2-week history of weakness and anorexia that was accompanied by a one-week history of fever. The patient was initially admitted to the Department of Respiratory Medicine Outpatient Clinic. Laboratory tests revealed a white blood cell (WBC) count of 10,750/μL, neutrophils (NE) of 9,130/μL, and C-reactive protein (CRP) levels exceeding 370 mg/L. Chest CT imaging demonstrated extensive consolidation in the right lower lobe of the lung along with right-sided pleural effusion. Despite antibiotic therapy, the patient’s fever persisted. Consequently, she was hospitalized for further diagnosis and treatment. The patient’s condition progressively worsened, and was manifested as blood-stained sputum, epistaxis, and bilateral eye congestion. Scleritis was subsequently diagnosed by an ophthalmologist. The patient had a medical history of chronic bronchitis. And the patient was not on any medication regimens. He has a long-standing smoking habit.

Vital signs were noted as follows upon admission: a peripheral pulse rate of 110 beats per minute, body temperature of 38.6°C, respiratory rate of 19 breaths per minute, and blood pressure of 110/66 mmHg. Physical examination did not find breath sounds in the right lower lung and revealed a soft abdomen with no palpable tenderness.

Laboratory findings were significant with a CRP level of 358.45 mg/L, white blood cell count of 13,020/μL, hemoglobin concentration of 10.7 g/dL, and platelet count of 623,000/μL. Urinalysis indicated the presence of proteinuria (1+) and abnormal red blood cell (205/uL) and white blood cell counts (60/uL), with 1–2 white blood cell casts per high-power field. The 24-hour urine protein excretion was measured at 1512 mg/day. Additional tests revealed a serum creatinine level of 65 *µ*mol/L, an estimated glomerular filtration rate (eGFR) of 106.33 ml/min/1.73 m^2^, and a serum albumin of 29 g/L. Procalcitonin (PCT) was 0.621 ng/ml, erythrocyte sedimentation rate (ESR) was 97 mm/h, and ANCA testing indicated a cANCA titer of 1:40 and PR3-ANCA of 988.8 CU.

**Figure 1:**
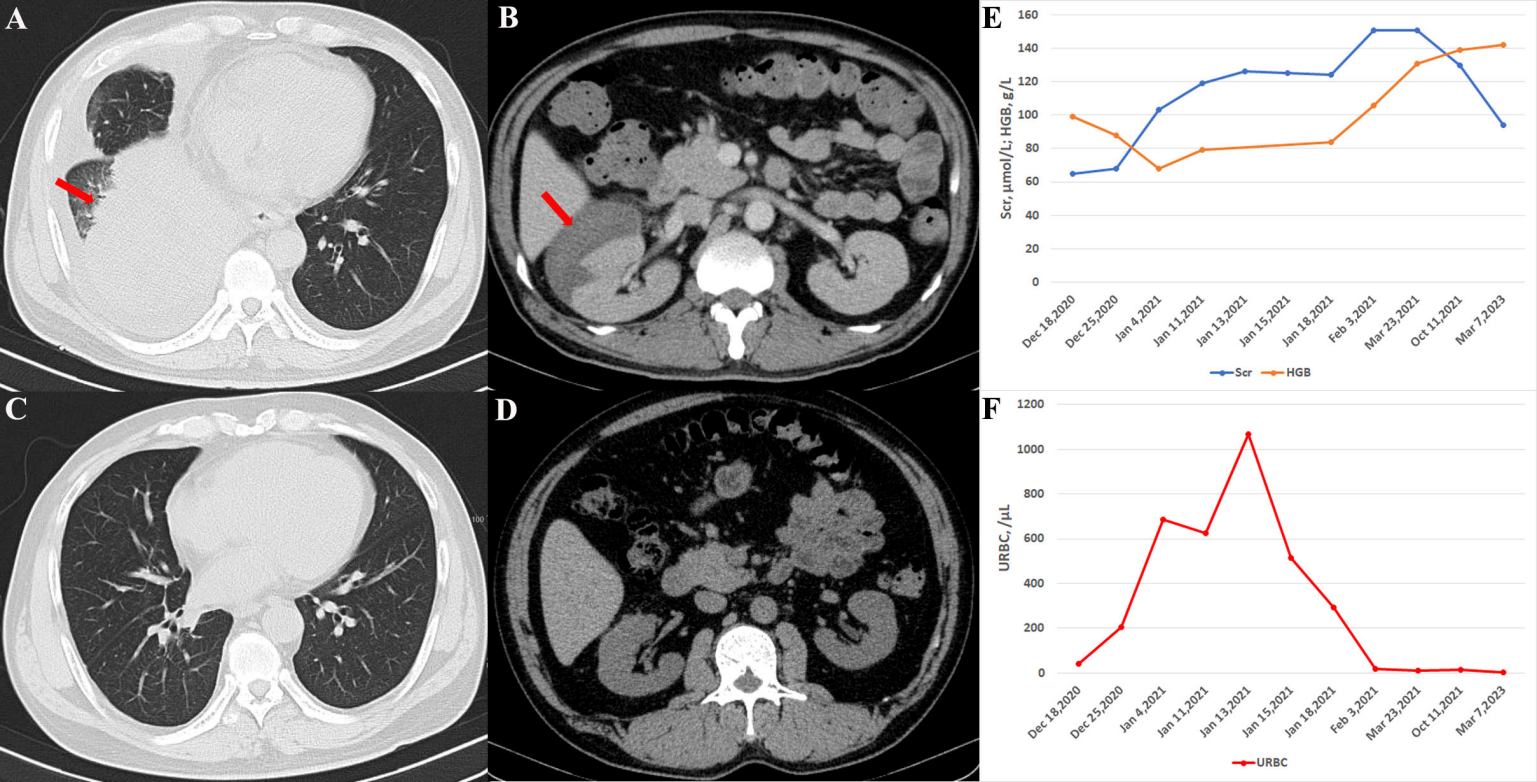
Imaging and laboratory testing of the patient. **(A)** Chest CT showing pulmonary consolidation (arrow) in the right lower lung upon admission. **(B)** The abdominal CT scan displays a large right-sided perirenal hematoma (arrow). **(C)** Six months later, the chest CT indicated complete resolution of right lung consolidation. **(D)** Perirenal hematoma disappeared six months after treatment. **(E)** Hemoglobin levels dropped to 68 g/L and serum creatinine rose to 103 *µ*mol/L on January 4, 2021, although both parameters gradually normalized following treatment. **(F)** Urine red blood cell count, which had significantly increased on January 4, 2021, also returned to normal after treatment.

**Figure 2:**
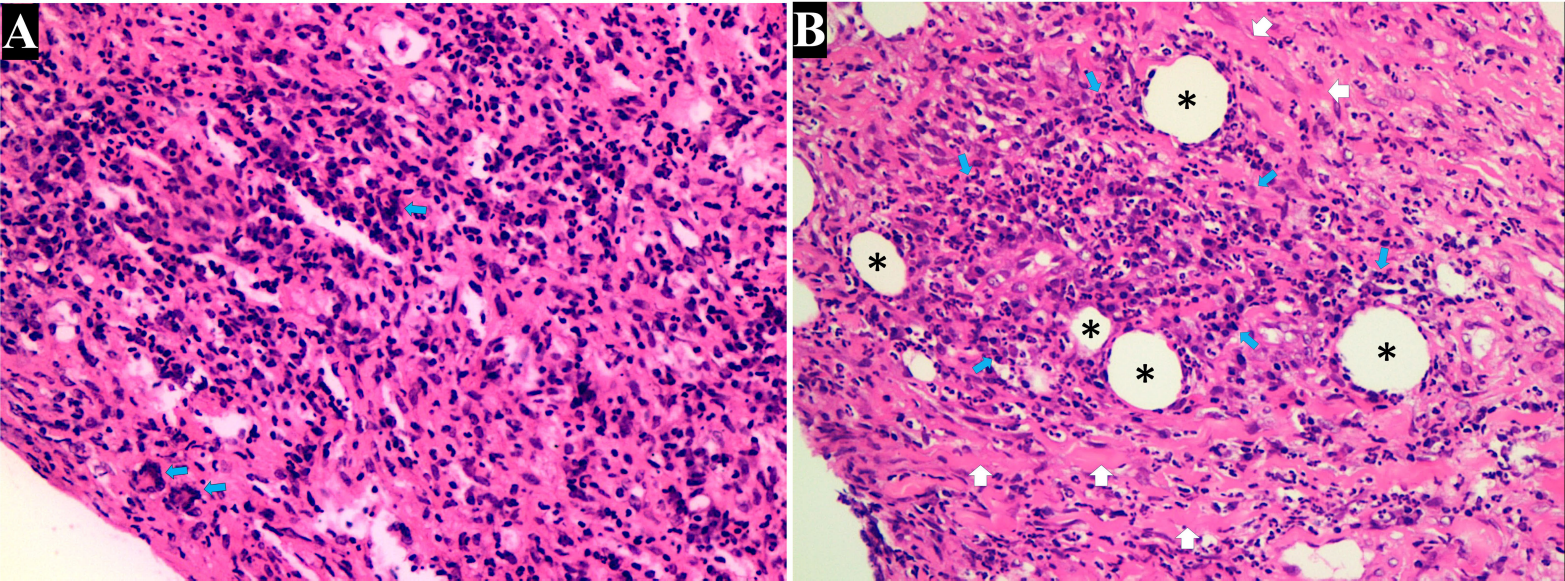
Histopathologic examination of the lung. **(A)** Histopathologic examination revealed extensive inflammatory infiltration, predominantly composed of lymphocytes and histiocytes interspersed within the connective tissue. Notably, the formation of granulomas (characterized by clusters of macrophages and the presence of multinucleated giant cells [blue arrow]), were indicative of granulomatosis with polyangiitis (H&E, 200×). **(B)** Advanced fibrotic changes with thick collagen deposits (white arrow) disrupted the normal tissue cytoarchitecture. Small, round spaces resembling blood vessels (black stars) suggested neovascularization amid the fibrotic tissue. Intense accumulation of lymphoplasmacytic cells (blue arrow) was observed around the small vessels (H&E, 200×).

An enhanced CT scan of the chest identified a large blood vessel that originated from the thoracic aorta and entered the lower lobe of the right lung, which we considered indicative of pulmonary sequestration alongside a mass in the lower lobe (**Figure 1A**). A CT-guided lung biopsy was performed, and the pathology report depicted multiple granulomas and multinucleated giant cell hyperplasia with inflammatory cell infiltration and focal necrosis in the blood vessel wall (**Figure 2**).

Based on the aforementioned laboratory tests, we established a diagnosis of GPA according to the 1990 American College of Rheumatology classification criteria (7). Initial treatment included methylprednisolone at a dosage of 80 mg/day was started.The patient’s fever was improved, but not completely resolved. No anticoagulant was used throughout the course of the disease.

Four days later, on 4 January 2021, the patient experienced sudden right abdominal pain which spontaneously resolved after one-half hour, but was accompanied by intermittent red urine. The patient reported no history of abdominal trauma. A repeat laboratory test showed a significant drop in hemoglobin from 10.7 g/dL to 6.8 g/dL. Other results comprised an elevated white blood cell count of 21,490/μL and a serum creatinine level of 103 *µ*mol/L, with a reduced eGFR of 71.61 ml/min/1.73 m^2^. A contrast-enhanced CT of the abdomen confirmed the diagnosis of SRH due to a subcapsular hemorrhage of the right kidney.

Given the active phase of GPA and persistent fever despite the current dose of methylprednisolone(80mg/d), we administered a higher pulse dose of methylprednisolone (250 mg/day for 3 days, followed by a taper), and rituximab was added to the regimen, with two doses of 500 mg given every 2 weeks. Following this treatment, the patient’s symptoms and laboratory parameters stabilized. The patient was prescribed oral prednisone at a dose of 80 mg/day following discharge, which was subsequently tapered gradually. The dose reduction protocol was to taper 5mg per week, to 40mg per day, then taper 5mg every 2 weeks, to 30mg per day, then taper 2.5mg every 2 weeks, to 15mg per day. Six months later, imaging studies showed resolution of the subcapsular hematoma and normalization of hemoglobin levels (**Figure 1**), although serum creatinine levels remained slightly elevated. The ANCA tests showed reduced titers (cANCA, 1:10 and PR3-ANCA, 26.2 CU). As of the final follow-up in March 2023, the patient’s condition remained stable. And the renal function, hemoglobin level, and urine red blood cell counts all returned to normal.

## Discussion

Spontaneous renal hemorrhage (SRH) is a rare, yet severe, complication of AAV, with a varied etiology that predominantly includes other forms of vasculitis such as PAN (4, 8). Despite the broader association with vascular diseases, the incidence of SRH specifically linked to GPA and MPA is notably infrequent. Our review and case study reflected on this rarity and emphasized the clinical vigilance required for the early detection and management of this group of diseases.

We conducted a comprehensive literature review of SRH secondary to AAV, spanning publications from 1 January 1990 to 1 April 2024. This search was performed using the PubMed database with keywords such as “ANCA-associated vasculitis,” “polyangiitis,” “granulomatosis,” “Wegener’s granulomatosis,” “granulomatosis with polyangiitis,” “microscopic polyangiitis,” “spontaneous renal hemorrhage,” “Wunderlich syndrome,” “subcapsular hematoma,” “perinephric hematoma,” and “spontaneous retroperitoneal hemorrhage.” This systematic search yielded 11 articles in which the authors collectively reported on 14 patients with SRH associated with AAV.

A total of 15 patients (including one case from the present study) were included in our statistical analysis (the clinical characteristics of these patients are summarized in **Table 1**). Of these, nine patients were diagnosed with GPA and six with MPA, at a ratio of 3:2. SRH was more prevalent in male patients (9), with a male-to-female ratio of 3:2; and we attributed this to the higher incidence of vascular aneurysms in males (3). The mean age of the patients was 54.5 years, and ranged from 25 to 82 years. The predominant clinical manifestation was acute flank pain that was observed in 100% of the cases, and was often accompanied by anemia and shock. The average white blood cell count was 20.0 ± 7.5 × 10^9^/L, and the average hemoglobin level was 67.0 ± 13.8 g/L. Other clinical manifestations included glomerulonephritis (78.6%), nodular lung infiltrates (58.3%), peripheral neuropathy (41.7%), and ears-nose-throat (ENT) involvement (25%). Treatment modalities varied. For example, eight patients received glucocorticoids and immunosuppressive agents that included rituximab, cyclophosphamide, and azathioprine; five underwent transcatheter arterial embolization; and one patient underwent nephrectomy. Two patients succumbed to complications, specifically complex infections and alveolar hemorrhage (9, 10). A comparative analysis between GPA and MPA patients revealed statistically significant differences in age and serum creatinine levels: GPA patients were younger than those with MPA (48.6 ± 12.4 vs. 63.5 ± 13.0, respectively; P = 0.043), and serum creatinine levels were significantly higher in MPA patients (P = 0.03).

**Table 1:** General characteristics of the patients.

	AAV (n/mean ± SD)	GPA (n/mean ± SD)	MPA (n/mean ± SD)	p
n	15	9	6	
Sex				0.237
Male	9	7	2	
Female	6	2	4	
Age(year)	54.5 ± 14.3	48.6 ± 12.4	63.5 ± 13.0	0.043
BVAS	21.0 ± 10.0	21.3 ± 2.4	20.7 ± 17.0	0.958
ESR(mm/h)	100.1 ± 35.0	88.7 ± 31.5	134.5 ± 21.9	0.111
CRP(mg/L)	183.7 ± 124.9	208.6 ± 102.6	158.8 ± 43.1	0.51
WBC (×10^9^/L)	20.0 ± 7.5	19.4 ± 8.4	21.4 ± 7.5	0.553
Hemoglobin (g/L)	67.0 ± 13.8	73 ± 15.7	62.7 ± 12.2	0.50
Serum creatinine(*µ*mol/L)	273.5 ± 192.8	205.3 ± 134.3	545.3 ± 149.7	0.03
Radiology				
Renal aneurysms, (n, %)	12 (100%)	7 (100%)	5 (100%)	
Organ involvement				
Kidney (n, %)	11 (78.6%)	8(88.9%)	3(60%)	0.560
Lung (n, %)	7 (58.3%)	5(71.4%)	2(40%)	0.621
ENT (n, %)	3(25%)	3(42.9%)	0	0.205
Peripheral neuropathy (n, %)	5(41.7%)	1(14.3%)	4(80%)	0.092
Treatment				
PSL (n, %)	13 (86.7)	8 (88.9%)	5(83.3%)	1.0
RTX (n, %)	2(16.7%)	2(28.6%)	0	0.470
CTX (n, %)	5(38.5%)	3(37.5%)	2(40%)	1.0
TAE (n, %)	5 (33.3%)	4(44.4%)	1(16.7%)	0.580
Nephrectomy (n, %)	1	0	1	0.400
Deaths (n, %)	2 (13.3%)	2 (22.2%)	0	0.483

SRH is typically manifested early in the disease course and is closely associated with disease activity (3, 10). In our analysis, 13 of the 15 patients experienced SRH within three months of the onset of their initial symptoms, corroborating findings from previous studies (3, 10). The remaining two patients developed SRH at 8- and 150-months post-symptom onset, respectively, and both underwent hemodialysis at that time (11, 12). This suggests that hemodialysis represents another risk factor for SRH, and that it is potentially exacerbated by the use of anticoagulant drugs during treatment. Furthermore, the Birmingham Vasculitis Activity Score (BVAS) exceeded 15 in five of the six patients, indicating that a majority were in an active stage of the disease. Commensurately, elevated ESR and CRP levels averaging 100.1 ± 35.0 mm/h and 183.7 ± 124.9 mg/L, respectively, were observed among these patients, underscoring the link between SRH and heightened disease activity.

AAV is a systemic vasculitis that primarily affects small-sized vessels, though medium-sized vessels can also be involved (1). Involvement of medium-sized renal vessels often leads to the formation of microaneurysms, and the subsequent rupture of these aneurysms is a primary cause of SRH in AAV (10–17). In the present study, all 12 patients who underwent computed tomography angiography (CTA) or arteriography were found to possess multiple renal aneurysms, as detailed in **Table 2**. The necrotizing inflammation characteristic of AAV can significantly weaken the vessel walls, predisposing them to aneurysm formation (11). Additionally, disruption of the internal and external elastic lamina constitutes another factor contributing to aneurysm development (18); and ultrasonography, CT, and angiography have been exploited to accurately identify and assess the presence of SRH (8).

**Table 2:** Clinical characteristic of SRH.

Studies	Diagnosis	Sex	Age (year)	Disease course (month)	BVAS	ESR (mm/h)	CRP (mg/L)	Treatment	Prognosis
Mengzhu Zhao (10)	GPA	M	64	<3	23	54	NA	TAE + MP (1g qd) + RTX	Died
	GPA	M	54		21	140	NA	MP (1g qd) + CTX	Relieved
	MPA	F	56		22	119	NA	MP (80 mg q12 h) + CTX	Improved
	GPA	M	44		23	101	NA	TAE + MP (1g qd)	Relieved
Yongquan Yu (13)	MPA	M	61	<3	NA	NA	NA	left nephrectomy	NA
Baker, 1978 (14)	GPA	M	25	1.5	NA	60	NA	TAE+CTX+PSL (30 mg/d)	Relieved
Nakashima (20)	MPA	F	77	3	NA	NA	114.3	PSL (30 mg/d)	Relieved
Taeko Ishii (21)	GPA	F	62	1	NA	NA	255	PSL (NA)+AZA	Relieved
Noriko Tamei (11)	MPA	M	55	8	3	NA	117	PSL (40mg/d)	Relieved
Raimund Senf (15)	GPA	M	35	2	NA	NA	NA	MP pulse+CTX	Relieved
Dhilip Andrew (17)	MPA	F	50	0.5	NA	150	NA	MP (NA)+CTX	NA
Ayumi Ishiwatari (16)	MPA	F	82	<3	37	NA	245	*PSL (40 mg/d)+TAE*	Relieved
Boersma (12)	GPA	F	51	180	NA	NA	NA	TAE	NA
Jin Tong (9)	GPA	M	50	1	NA	80	12.36	PSL (0.6 mg/kg/d) +CTX	Died
This study	GPA	M	52	2	23	97	358.45	MP (200 mg/d)+RTX	Relieved

GC, glucocorticoid; MP, methylprednisolone; PSL, prednisolone; TAE, transcatheter arterial embolization; RTX, Rituximab; NA, not available; F, female; M, male; ENT, ears-nose-throat; CTX, cyclophosphamide; AZA, azathioprine; RTX, rituximab.

The treatment of SRH varies considerably based on its etiology. For SRH associated with tumors, radical or partial nephrectomy or embolization is typically required (4, 19). However, SRH in patients with AAV often responds well to non-surgical treatments, including glucocorticoids and immunosuppressants. Management strategies for AAV-induced SRH should therefore include supportive measures such as aggressive fluid resuscitation and blood transfusion. The use of glucocorticoids and immunosuppressants is generally guided by the overall activity of the disease, and in cases where there is no clear evidence of systemic disease activity, a less intensive treatment regimen may suffice for managing renal hemorrhage.

The patient described in this study experienced remission of abdominal pain, stabilization of vital signs, and cessation of hemoglobin decline following treatment with pulse glucocorticoids. Consequently, further selective arteriography and embolization were deemed unnecessary. In our analysis, 53.3% of the patients relieved after conservative treatment. Four patients received steroid pulse therapy, and two of these patients also required renal artery embolization (10). Three patients additionally developed SRH within one to three weeks of initiating steroid pulse therapy due to the involvement of other vital organs (11, 15, 16). Conventional doses of corticosteroids and immunosuppressants were successfully employed to treat four patients (10, 17, 20, 21). However, continuous monitoring of vital signs is essential; and for those individuals showing persistent deterioration despite conservative measures, we recommend angiography to identify actively bleeding vessels, followed by necessary embolization.

Of the 15 patients reviewed with SRH, five underwent angioembolization, and one patient required nephrectomy. One MPA patient did not respond to conservative treatment, and this led to examination by arterial angiography that revealed active bleeding from the superior segmental renal artery, which was subsequently embolized (16). The use of heparinization therapy in this case might explain the failure of the conservative approach. Boersma et al. reported on a patient diagnosed with GPA 15 years earlier and who was dependent upon hemodialysis (12). Rapid blood transfusion and fluid resuscitation were insufficient to stabilize this patient. Subsequent selective catheterization of the right renal artery showed clear extravasation of contrast media from an aneurysm, leading to targeted embolization of the affected aneurysms. We posit that renal replacement therapy and the use of anticoagulant drugs may ultimately pose significant risks for the failure of conservative treatments.

## Conclusions

In this study, it was found that SRH represented a rare but significant complication of AAV that is typically manifested early in the disease course and that is closely linked to disease activity. The primary cause of SRH in AAV patients was the rupture of renal aneurysms; therefore, vigilant monitoring is crucial for managing patients with SRH. Moreover, if conservative treatments are ineffective, selective arterial embolization should be considered as a critical intervention to prevent more drastic measures such as nephrectomy, and also to mitigate the risk of mortality.

## Data Availability

The original contributions presented in the study are included in the article/**Supplementary Material**. Further inquiries can be directed to the corresponding author.
