# Supplementary material for: Case report: Spontaneous renal hemorrhage in anti-neutrophil cytoplasmic antibody-associated vasculitis
Source: Front Immunol. 2025 Jan 29;16:1544263. doi: 10.3389/fimmu.2025.1544263 (PMC11813874; doi:10.3389/fimmu.2025.1544263)
Supplement: Supplementary file 1 [file DataSheet1.docx]

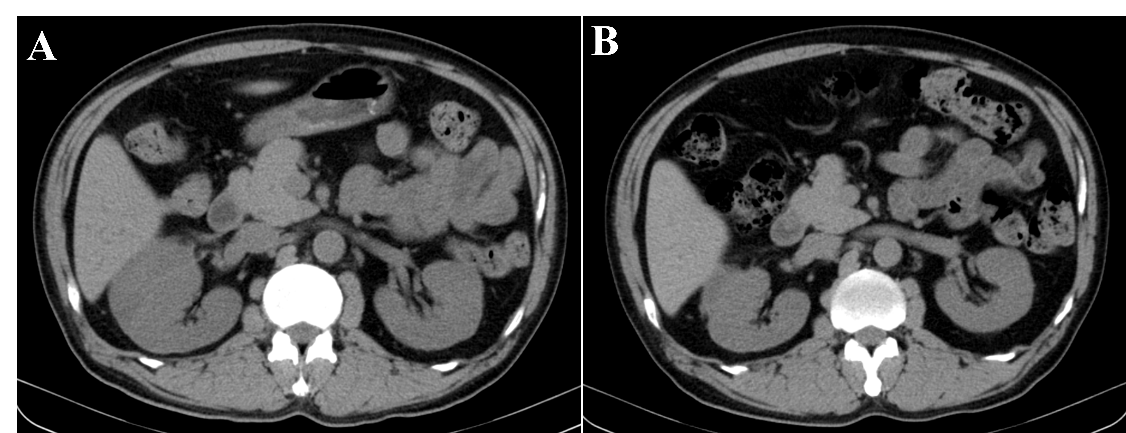


Supplementary figure1 The abdominal CT scan of the patient

1. One month after treatment, the abdominal CT indicate that the right-sided perirenal hematoma was smaller. B. After a two-month period, the perirenal hematoma significantly decreased.

Supplementary Table 1 laboratory testing of the patient during follow up

| Date | Scr(μmol/L) | HGB(g/L) | URBC(n/μL) |
| --- | --- | --- | --- |
| Dec 18, 2020 | 65 | 99 | 43 |
| Dec 25, 2020 | 68 | 88 | 205 |
| Jan 4, 2021 | 103 | 68 | 685 |
| Jan 11, 2021 | 119 | 79 | 625 |
| Jan 13, 2021 | 126 | NA | 1067 |
| Jan 15, 2021 | 125 | NA | 514 |
| Jan 18, 2021 | 124 | 84 | 292 |
| Feb 3, 2021 | 151 | 106 | 20 |
| Mar 23, 2021 | 151 | 131 | 12 |
| Oct 11,2021 | 130 | 139 | 15 |
| Mar 7, 2023 | 94 | 142 | 3 |

NA: not available
